# Role of Uric Acid in Semen

**DOI:** 10.3390/biom8030065

**Published:** 2018-07-31

**Authors:** Saleem Ali Banihani

**Affiliations:** Department of Medical Laboratory Sciences, Jordan University of Science and Technology, Irbid 22110, Jordan; sabanihani@just.edu.jo; Tel.: +962-2-720-1000

**Keywords:** uric acid, sperm, semen quality, antioxidant

## Abstract

Since 1963, various research studies and reports have demonstrated the role of uric acid (2,6,8-trihydroxypurine), an end product of adenosine and guanosine catabolism, on semen quality and sperm function. However, this effect has not yet been collectively discussed, even though uric acid has been a well-recognized constituent in semen. Here, we systematically and comprehensively discuss and summarize the role/effect of uric acid in semen quality by searching the main databases for English language articles considering this topic. Additionally, certain significant and relevant papers were considered to support discussions and perceptions. In conclusion, uric acid contributes to maintaining and enhancing sperm motility, viability, and morphology; therefore, protecting sperm function and fertilizing ability. This contribution is performed mainly by neutralizing the damaging effect of oxidizing (e.g., endogenous free radicals and exogenous toxins) and nitrating agents and enhancing certain bioactive enzymes in spermatozoa. In contrast, high levels of uric acid may induce adverse effects to sperm function, at least in part, by reducing the activity of vital enzymes in spermatozoa. However, further research, mainly clinical, is still required to fully explore the role/effect of uric acid in semen.

## 1. Introduction

**Figure 1:**
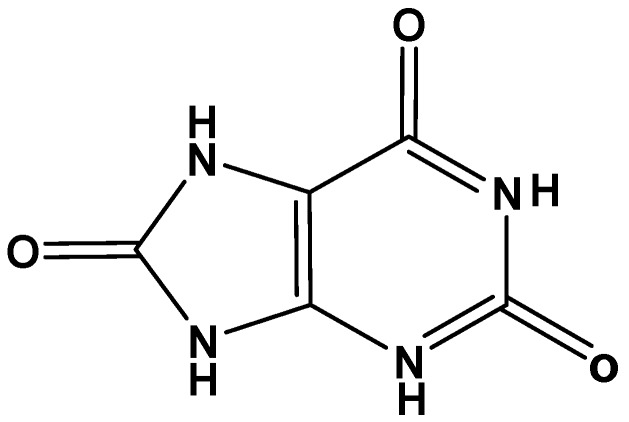
Chemical structure of uric acid.

Uric acid (C_5_H_4_N_4_O_3_; 2,6,8-trihydroxypurine), an inert heterocyclic molecule (Figure 1), is the end product of purine nucleotides (guanosine and adenosine) catabolism in humans and higher primates [1]. Mammals and lower primates other than humans carry purine catabolism one step further with the formation of allantoin from urate, a step catalyzed by uricase, which is an oxidoreductase enzyme [2]. At physiologic pH, uric acid is usually presents as its monoanion urate, which is neutralized by sodium cations. Among the total non-protein nitrogen compounds in human plasma and urine, urate forms approximately 10%, and 1.7%, respectively [3].

In the human body, at 37 °C and 7.4 pH, urate is relatively soluble; therefore, supersaturation of sodium urate in the blood leads to its crystallization and precipitation in joints. This precipitation may lead to a form of inflammatory arthritis known as “gout”. Usually, such conditions can occur because of excess dietary protein intake, underexcretion of urate by the kidney, and inherited metabolic disorders.

Various studies have shown that uric acid is a potent antioxidant, capable particularly of neutralizing hydroxyl radicals (^•^OH) and hypochlorous acid (HOCl), before it is converted to innocuous products such as allantoin, glyoxylate, allantoate, urea, and oxalate [1]. In addition, urate was found to serve as a coenzyme for certain oxygenase enzymes. Moreover, interestingly, studies have shown that urates prevent inactivation of certain endothelial enzymes such as angiotensin-converting enzyme and cyclooxygenase enzyme [1].

Since 1963, various studies (direct, indirect, basic, and clinical) have revealed the role of uric acid in semen quality; this role, though, has yet to be collectively summarized. Here, we systematically and comprehensively discuss and summarize the role/effect of uric acid on semen quality. To undertake this, we searched the PubMed, Scopus, and Web of Science databases for English language papers (fully-published or abstracts) from July 1963 to June 2018 using the key words “uric acid” or “urate” vs. “sperm”. In addition, certain significant and relevant papers were considered to support the observed mechanistic perceptions and discussions.

## 2. Uric Acid as a Component of Semen

In general, non-protein nitrogen compounds, including uric acid, are present in semen [4,5,6]. They are possibly derived from two sources: transudation from the blood circulation [4] or local production [4,7]. In bulls, urate is probably produced by oxidation of xanthine and hypoxanthine in the seminal vesicles [8]. In certain cases, such as retrograde ejaculation, the sperm come into direct contact with uric acid present in urine, which might increase to toxic concentrations [4].

A number of animal studies revealed that certain physical, social, and environmental factors may influence the level of seminal urate. For example, a study on cockerels showed that the concentration of uric acid in seminal plasma may be affected by the semen collection method [9]. In addition, the mean value of seminal urate was found to be higher in younger bulls compared to old ones [10]. Moreover, uric acid in semen was found to be affected by temperature; its level was found to be higher in cold conditions compared to warm conditions [10]. 

Alternatively, in vitro short-term storage of sterlet (*Acipenser ruthenus*) semen, at 4 °C for 36 h, was found not to change the level and the bioactivity of uric acid [11]. Also, uric acid was unaffected by restricted diet in male collared peccaries [12].

Urate displayed approximately equal or slightly elevated concentrations compared with serum [13]. The concentration ratio of serum urate to seminal plasma urate is approximately one [13]. The content of urate in seminal plasma in normozoospermia (*n* = 44), oligozoospermic (*n* = 19), and azoospermic (*n* = 10) men were 0.47 ± 0.13 mmol L^−1^, 0.48 ± 0.23 mmol L^−1^, and 0.45 ± 0.14 mmol L^−1^, respectively [13].

## 3. The Antioxidant Activity of Uric Acid in Semen

Urate is one of the major antioxidants in human semen, and its antioxidant activity in semen contributes almost half as much as ascorbate [14]. It has been shown that the antioxidant activity of uric acid against reactive oxygen species is mostly restricted to the semen, with little activity in the epididymis [14]. Various components of seminal plasma, including urate, contribute to its fast total radical-trapping antioxidant potential. It has been shown that 37% of the antioxidant activity in semen is attributed only to ascorbate, urate, and tyrosine [15]. In other species such as brown trout, a European species of salmonid fish, urate was found to be the main seminal antioxidant [16].

Lower levels of urate might be considered as a toxicity marker from exogenous oxidants such as methyl parathion, an organophosphorus pesticide, in testis [17,18], epididymis [19,20], and seminal plasma [21]. In addition, it has been shown that the lower urate concentrations found in seminal fluid of infertile patients might be indicative of decreased anti-oxidative protection, which could be dangerous to sperm integrity [22].

## 4. Effect of Uric Acid on Sperm Parameters

Table 1 summarizes the research studies that reveal the direct effects of uric acid on semen quality and sperm parameters. Almost all main sperm parameters such as motility, count, morphology, and DNA damage are significantly affected by seminal uric acid, showing improved sperm parameters at normal and even at higher seminal uric acid concentrations. In contrast, urate crystals were detected in semen of a patient with symptoms of chronic prostatitis, and these crystals could be behind the observed sperm abnormalities [23].

Various research studies have investigated the level of uric acid in different infertile men groups. It was found that mean concentrations of uric acid for normal men are higher than for those men with azoospermia and oligozoospermia [28]. In addition, among the infertile groups, azoospermic men had the lowest seminal uric acid levels [29]. Also, normozoospermic patients had lower urate concentrations (320 ± 22 µM) compared with healthy donors (426 ± 26 µM) [22]. This evidence, in general, shows that urate levels in seminal plasma are lower in infertile men compared to that of fertile men.

## 5. Mechanistic Studies 

The role of uric acid in preserving and/or improving sperm parameters such as motility, viability, morphology, and DNA integrity, and hereafter increasing the fertilizing ability of sperm, is due principally to its antioxidant potential.

Sperm generate small amounts of nitric oxide (NO) and superoxide ion (O_2_^•−^). The reaction between these two radical species results in formation of peroxynitrite (ONOO^−^) [30]. This reaction is very fast (6.7 × 10^9^ mol s^−1^) and irreversible due to its exothermic nature [30,31]. Peroxynitrite is a strong oxidizing and/or nitrating agent [32,33]. It can attack all main biomolecules (DNA, proteins, lipids) in sperm [34,35]. In addition, ONOO^−^ may react with the tyrosine moieties in any given cellular protein and form 3-nitrotyrosine, a nitrated product and biomarker [33]. Such oxidation and/or nitration reactions may lead to sperm injury and sperm death [35]. Uric acid was found to be the most effective scavenger of ONOO^−^ in spermatozoa, which enhances its function, and hence the fertilizing capability [36,37]. 

Moreover, the hydroxyl radical (^•^OH) and O_2_^•−^ were found to reduce human sperm motility [38], viability [39], and morphology [40]. In cellular systems, ^•^OH is generated from hydrogen peroxide (H_2_O_2_) when reacted with O_2_^•−^ in the presence of reduced forms of metal ions such as ferrous (Fe^2+^) and cuprous (Cu^+^) ions [41,42,43]; such reactions are known as Fenton’s reactions. Studies demonstrated that urate is able to scavenge both ^•^OH and O_2_^•−^ radicals and protect against oxidative damage, particularly DNA damage and lipid peroxidation [44,45,46]; thus, enhancing sperm function, and consequently the fertilizing ability.

Other important functions for seminal uric acid is that it enhances certain bioactive enzymes that are considered crucial for adequate sperm function.

Cyclooxygenase, also called prostaglandin-endoperoxide synthase, is an enzyme responsible for synthesis of prostanoids such as prostaglandins and thromboxane. Cyclooxygenase 1 (COX-1) and cyclooxygenase 2 (COX-2) isoforms are present in human semen and involved in sperm function and the fertilization process [47]. It has been shown that urate serves as a coenzyme for cyclooxygenase [1]. Studies have revealed that urate, at its physiological level, prevents oxidative inactivation of cyclooxygenase [1,48]. Accordingly, this may be valuable to enhance sperm function and fertilization rate. In fact, urate was found to have a role in the conversions from biologically inactive precursors into active prostaglandin derivatives in the accessory organs of reproduction [7,13,49].

In contrast, nitric oxide, a free radical gas, is produced from L-arginine by a class of enzymes known as nitric oxide synthases (NOS) [50]. It has been shown that a NOS similar to endothelial nitric oxide synthase (eNOS) and brain nitric oxide synthase (bNOS) are present in human spermatozoa [50]. Nitric oxide was found to be crucial for adequate motility of human spermatozoa [51,52,53]. Therefore, decreased levels of nitric oxide, below normal, reduce sperm motility [54]. High concentrations of urate (12 mg dL^−1^, for 24 h treatment) significantly attenuated eNOS activity, and thus nitric oxide production in human umbilical vascular endothelial cells by decreasing the binding between calmodulin and eNOS [55]. Accordingly, it is acceptable to suggest that high levels of uric acid in human semen may reduce sperm motility and function, and direct experimental studies to approve this suggestion seem valuable.

In addition, adenosine triphosphate (ATP) is generated in human spermatozoa from the chemical shuttle between creatine, a nitrogenous organic acid synthesized in the liver from the amino acids arginine and glycine, and creatine phosphate, which is catalyzed by creatine phosphokinase (CPK), also called creatine kinase (CK) [56]. High concentration of urate (~6.8 mmol L^−1^) was found to inhibit all isoforms of creatine kinase (CK-MM, CK-MB, CK-BB) and may be by affecting the SH groups of the enzyme [57,58]. Therefore, high concentrations of urate in semen may decrease sperm function, particularly sperm motility, by inhibiting the activity of CK, and as a result, reducing the normal levels of chemical energy produced. It is important to point out that the increased levels of uric acid in semen is an abnormal condition that occurs only at certain occasions such as retrograde ejaculation, when the sperm come into direct contact with uric acid present in urine, or maybe in the presence of very high levels of uric acid in the blood, which is not yet completely approved [4]. 

The correlation between uric acid and other bioactive molecules/ions in semen was explored. For example, urate displayed inverse relationships against seminal fructose [13]. In addition, seminal urate and xanthine correlated significantly to each other in oligozoospermic and normal men [13].

Moreover, healthy fertile men with high magnesium concentrations (3.43 ± 1.77 mmol L^−1^) had lower seminal uric acid levels compared to those with low magnesium concentrations (1.53 ± 0.39 mmol L^−1^) [59]. The possible explanation for such correlation is that higher magnesium concentrations may enhance sperm metabolism resulting in increased production of reactive oxygen species [59]. These pro-oxidants oxidize uric acid and decrease its antioxidant activity. Indeed, total antioxidant capacity was found to be lower in men with high seminal magnesium concentrations compared to those with low magnesium concentrations [59].

Another possible explanation for the correlation between magnesium and uric acid is that higher magnesium levels in semen may accelerate sperm metabolism resulting in increased formation of reactive oxygen species [59]. Further, a positive relationship was found between magnesium and interleukin-12, which is noted to have a pro-inflammatory property, and may contribute to the increased total oxidation status values.

The variation between uric acid levels in blood and uric acid in semen was investigated, but not in profound detail. There were no significant differences in the level of uric acid in both seminal plasma and blood between subjects with normozoospermia and patients with abnormal semen parameters (e.g., oligoasthenozoospermia, asthenozoospermia, oligozoospermia, and cryptozoospermia) [60]. Such results are indicative of the equal importance of uric acid in semen and blood. In fact, the reabsorption mechanism of uric acid in kidneys is indicative of the importance of uric acid in the body; almost all (>99%) filtrated uric acid by the glomerulus is reabsorbed in the proximal tubule back to the blood [61]. 

## 6. Conclusions and Future Perspectives

In summary, uric acid contributes to preserving and enhancing sperm motility, viability, and morphology, which in turn protects sperm function. This contribution is achieved principally by counteracting the damaging effect of oxidizing (e.g., reactive oxygen species and toxins) and nitrating agents. In addition, uric acid enhances certain bioactive enzymes that are vital for sperm function. In contrast, in certain circumstances, high levels of uric acid in semen may induce adverse effects to sperm function, this may occur by the reduction of the activity of vital seminal enzymes such as eNOS and CK. Additional research studies, mainly human studies, are still required to fully investigate the role of uric acid in semen and sperm function. 

## Figures and Tables

**Table 1:** Studies that demonstrate the direct effect of uric acid on semen and sperm parameters.

Affecter	Type of Study	Study Population/Sample	Effect on Sperm Parameters	Ref.
**Uric acid**	In vitro	Human sperm	(+) Sperm motility	[4]
**Urate at 400 µM**	In vitro	Human sperm	(−) X-ray-induced sperm DNA damage	[24]
**(+) Seminal uric acid**	Observational	Human semen and sperm	(+) Sperm motility (+) Sperm morphology (±) Sperm concentration (±) Semen pH (±) Semen volume	[25]
**Uric acid at (0.25, 0.5/5–7 × 10^7^ cells mL^−1^)**	In vitro	Brown trout semen	(+) Sperm membrane integrity (+) Sperm motility (−) Sperm lipid peroxidation	[16]
**(+) Seminal uric acid**	Observational	Sub-fertile men	(±) Semen volume (±) Sperm count (±) Sperm motility	[26]
**Uric acid at 0.25 mmol L^−1^**	In vitro	Cryopreservation extenders of rainbow trout	(+) Sperm motility	[27]
**(+) Seminal uric acid**	Observational	Human semen	(+) Sperm concentration	[28]

(−) decrease; (+) increase; (±) no effect.
